# Community Rehabilitation and Hospitalizations Among People With Chronic Psychotic Disorder: Is There a Differential Association by Co-occurring Substance Use Disorder?

**DOI:** 10.3389/fpsyt.2021.621259

**Published:** 2021-02-05

**Authors:** Sharon Florentin, Paola Rosca, Tali Bdolah-Abram, Yehuda Neumark

**Affiliations:** ^1^The Hebrew University Hadassah Medical School, Faculty of Medicine, Hebrew University of Jerusalem, Jerusalem, Israel; ^2^Department for the Treatment of Substance Abuse, Ministry of Health and The Hebrew University of Jerusalem, Jerusalem, Israel; ^3^Faculty of Medicine, The Hebrew University of Jerusalem, Jerusalem, Israel; ^4^Hebrew University-Hadassah Braun School of Public Health & Community Medicine, The Hebrew University of Jerusalem, Jerusalem, Israel

**Keywords:** schizophrenia, substance use disorder, rehabilitation, co-occurring disorders, hospitalizations, Israel

## Abstract

**Objective:** Co-occurrence of chronic psychotic disorders and substance use disorder (SUD) is clinically challenging and increasingly prevalent. In 2000, legislation was passed in Israel to foster rehabilitation and integration in the community of persons with mental health disorders. In 2010, the need to allocate resources for patients with these co-occurring disorders (COD) was officially recognized. Yet, most rehabilitation services were not specifically designed for COD. This study examines the relationship between duration of community rehabilitation and number of psychiatric hospitalization days among persons with/without COD in Israel.

**Methods:** Data from the National Psychiatric Case Register on 18,684 adults with schizophrenia/schizoaffective disorders hospitalized in 1963–2016, was merged with data from the Israel Mental Rehabilitation Register. Associations and interactions between COD-status (COD/non-COD), time-period (Period_1_: 2001–2009, Period_2_: 2010–2016), duration of housing or vocational rehabilitation on hospitalization days per year were analyzed using repeated-measures ANOVA.

**Results:** The proportion of non-COD chronic psychotic patients who received rehabilitation services increased from 56% in Period_1_ to 63% in Period_2_, as it did among COD patients—from 30 to 35%. The proportion of non-COD patients who received longer-duration vocational rehabilitation (≥1 year) was significantly higher (43%) than among COD patients (28%) in both time periods. For housing rehabilitation, these proportions were 79 and 68%, respectively. Persons with COD experienced more hospitalization days annually than non-COD patients. Duration of rehabilitation (less/more than a year) was inversely associated with annual number of hospitalization days (*p* < 0.0001). This pattern was noted in both COD and non-COD groups and remained significant after controlling for age, sex, COD group, percent of hospitalizations with SUD, and age at first hospitalization.

**Conclusions:** COD patients with prolonged rehabilitation seemingly achieve long-term clinical improvement similar to non-COD patients, despite most rehabilitation settings in Israel not being designed for COD patients. Yet, COD patients receive overall less rehabilitation services and for shorter periods than non-COD patients. Long-term rehabilitation services should be provided to COD patients, who may need more time to commit to treatment. To achieve better long-term mental health improvements, a continued expansion of community-based integrative treatment and rehabilitation services for COD patients is needed in Israel.

## Background

One-fourth to two-thirds of patients with schizophrenia in the US and in Europe have a co-occurring Substance Use Disorder (SUD) (1–6). Analyzing data from the Israel National Psychiatric Case Register (INPCR), we recently reported a co-occurring disorder (COD) rate of 35% among Israeli adults with schizophrenia or schizoaffective disorder (7).

The co-occurrence of schizophrenia/schizoaffective disorder and SUD (also referred to as “dual diagnosis”) is often characterized by a chronic relapsing course of illness. The recurrence or worsening of the SUD can also trigger relapse of a psychotic episode (8). SUD worsens the overall clinical course of schizophrenia—compared to people with schizophrenia only, people with co-occurring SUD tend to be less adherent to treatment, experience more frequent relapses, and have higher rates of violent and life-threatening behavior, suicides and homelessness (9–12). The treatment of persons with COD is particularly challenging and often more complex than treatment of persons without COD (13). In early stage disease (i.e., within 5 years from initial diagnosis), COD patients show fewer brain deficits than non-COD patients, however, over time, the clinical picture is reversed and brain deficits such as volume deficits, shape abnormalities, and abnormalities in default mode network activation are more commonly manifested among COD patients (14, 15). This change is related to the long-term neurotoxic sequelae, but still on average COD patients have more preserved social and emotional functions during the premorbid phase as well as later on. COD patients also have better executive functioning which enables them to maintain their substance using behaviors (14).

The positive effect of community rehabilitation services on patients with severe mental illness, and schizophrenia in particular, has been widely reported (16–19). Community rehabilitation helps improve the functioning, well-being, symptoms severity and self-esteem of schizophrenia patients. There is also worldwide evidence that community rehabilitation is associated with a reduction of hospitalizations and frequency of hospitalization among persons with schizophrenia (20–23). This is likely achieved through positive effects of employment and regular contact and monitoring of the patient which allows for early detection of deterioration in the individual's mental health status and rapid referral for primary care thereby precluding the need for hospitalization.

The *Community Rehabilitation of Persons with Mental Disability Law* was enacted in Israel in the year 2000 with the aim to foster “the rehabilitation of persons with mental health disorders and their integration in the community, in order to enable them to attain the maximal degree of functional independence and quality of life, while maintaining their dignity.” This law initiated a reform in hospitalization services with the aim of reducing the number of psychiatric hospital beds alongside the establishment of community rehabilitation services, which began operating in 2001.

Of the rehabilitation services offered (vocational, housing, educational, leisure, family support, and treatment coordinator), vocational and housing rehabilitation are the most common, offer the greatest support and are usually the longest in duration. The aim of housing rehabilitation is to enhance social and housekeeping skills for independent-living in the community, by means of finding suitable and dignified housing conditions, with provision of support and on-going contact with community services. Vocational rehabilitation services promote finding and maintaining employment adapted to the wishes and capacities of the individual (24). Rehabilitation programs are generally personalized and accompanied by mental health therapists.

Until recently no rehabilitation services were available for persons with co-occurring disorders of severe mental illness (SMI) and SUD, and those with active drug use were generally denied services. It was only in 2010 that the Israeli parliament officially recognized the need to allocate additional resources for patients with COD (25). These resources included gradual opening of services for COD patients in hospitals and in the community and training of mental professionals about COD.

Enactment of the Community Rehabilitation law lead to the opening of private community-based rehabilitation services regulated and funded by the Ministry of Health (26) and was the precursor of the national insurance mental health reform formally launched in 2015. The reform aimed to integrate mental health services into the general healthcare system (primarily provided by the four HMOs that serve the Israeli population), and reduce the number of psychiatric hospitalizations through the expansion of ambulatory services (27). Overall, these policy shifts have resulted in the reduction of psychiatric hospital beds, number of hospitalization days, and rate of psychiatric hospitalizations (24, 27). People with SMI receiving rehabilitation are being hospitalized for shorter periods and the time between hospitalizations has lengthened (28). This was also true for schizophrenia and schizoaffective disorder patients in particular (22). The re-hospitalization rate decreased among patients with schizophrenia with an in-patient stay longer than 6 months (chronic patients) although not for short-stay patients with schizophrenia or affective disorders (29).

Regrettably, the treatment of addictions and the care of COD patients of SMI and SUD were excluded from the mental health reform of 2015. Patients with COD remained under the responsibility of the Ministry of Health, and are not entitled to receive treatment for their disorders from their HMO (27). Thus, the therapeutic options offered to COD patients, both in hospitals and in the community were, until very recently, limited, and many patients remained without adequate treatment (30–32). This situation existed, despite a growing awareness in recent years among health policy-makers in Israel about the need to allocate additional resources to treat patients with COD (25).

The present study, the first in Israel, examines the relationship between mental rehabilitation in the community and hospitalization characteristics of people with chronic psychotic disorder with and without SUD. We hypothesized that the rate of COD (comorbid chronic psychotic disorders and SUD) patients receiving rehabilitation services will be lower than that of non-COD patients, and that rehabilitation will be associated with a decrease in the number of hospitalization days over time for both groups, with a more substantial decrease among those without SUD.

## Methods

Data were extracted from Israel's Mental Rehabilitation Register (IMRR) and merged with the National Psychiatric Case Register (NPCR) of the Ministry of Health. The NPCR is the official register of all psychiatric admissions and discharges countrywide since 1950 (33). As described in a previous report (20), we identified all adult patients (aged 18–65) hospitalized in a psychiatric hospital or a psychiatric ward of a general hospital during the period 1963–2016, with an ICD-10 diagnosis (34) of schizophrenia (F20) or schizoaffective disorder (F25) (SZ-SAD) at their last discharge. We restricted the study population to persons hospitalized at least once during the years 2010–2015 in order to ensure that the data and the findings are relevant.

For each hospitalization, a SUD diagnosis is recorded based on an ICD-10 diagnosis of F10–F19 in the categories of dependence and abuse (excluding F17—nicotine dependence) and/or a psychiatrist-documented indication of alcohol and/or drug abuse at admission or discharge. Patients were classified as COD if they had a SUD diagnosis in two or more hospitalizations, or in at least 20% of their hospitalizations.

Each person's hospitalization history was documented from his/her first hospitalization until the end of 2016. A total of 18,684 patients with 168,377 hospitalizations were included in the analysis after exclusion of 29 patients who had an anomalous number of hospitalizations (≥80).

The hospitalization data was merged with data from the IMRR that included: applications to the regional rehabilitation committee, type of rehabilitation service approved (either housing or vocational) and the total length of time the person actually received the service in each of the two time periods 2001–2009 (Period_1_) and 2010–2016 (Period_2_). We divided the periods before and after 2010, the year in which the Israeli parliament officially recognized the need to allocate additional resources to treat patients with COD (25). Community rehabilitation services began operating in 2001, and hence this defined the start of the study period.

To assess the impact of rehabilitation duration on hospitalization patterns, we compared hospitalization patterns of people who did not receive any rehabilitation services, people who received rehabilitation services for <1 year and those who received services a year or more. A one-year cutoff was adopted because the likelihood of dropping out of rehab is greatest during the first year (29, 35) and often is the result of the person not fitting in (e.g., not allowing therapeutic contact) or not complying with the service's rules (e.g., harassing or assaulting other service recipients). Completion of the first year often suggests that the person is benefiting from the service in terms of improved quality of life, clinical condition and social functioning, and rehabilitation is likely to be sustained for longer periods of time (28, 35, 36).

This secondary analysis study was conducted in accordance with the Helsinki Declaration and was approved by the IRB of the Israel Ministry of Health. Patients' identification information was anonymized from all datasets prior to being released to the researchers.

### Analysis

Associations between the independent variables [COD-status and length of housing/vocational rehabilitation in Period_1_ (2001–2009) and in Period_2_ (2010–2016)] and mean annual number of hospitalization days were assessed using χ^2^ and *t*-tests, as appropriate. Repeated measures ANOVA was used to track changes in hospitalization variables across the two time periods. Multivariate ANCOVA modeling for mean hospitalization days per year was performed to identify predictors including rehabilitation length, age, age at first hospitalization, sex, COD-status and percent of hospitalizations with SUD. Statistical significance level was set at *p* < 0.05 to help guide interpretation of the results. The data were analyzed using IBM® SPSS® Statistics, version 24.0.

## Results

Of the 18,684 persons with a psychiatric hospitalization between 2001 and 2016, 28.8% had a co-occurring disorder (Table 1). The COD group was predominantly male (85%) compared with 58% of the non-COD group, and was 3.5 years younger, on average, than the non-COD group. For persons with COD age at first hospitalization was 2.4 years younger than those without COD. The rate of approval by the regional rehabilitation committees for housing rehab services was slightly higher among those with chronic psychotic disorder without COD (90%; SD = 23%) than among persons with COD (85%; SD = 27%) (*p* < 0.0001). The rate of vocational rehabilitation service approval was identical in both groups (93%).

**Table 1:** Demographic characteristics of people with schizophrenia/schizoaffective disorder (aged 18–65) with a psychiatric hospitalization during the period 1963–2016, by co-occurring SUD status, Israel.

	**Total**		**COD**		**Non-COD**	
	**%**	**No**.	**%**	**No**.	**%**	**No**.
No.	100	18,684	28.8	5,379	71.2	13,305
**Sex**						
Female	34.2	6,387	15.2	818	41.9	5,569
Male	65.8	12,297	84.8	4,561	58.1	7,736
**Population group**						
Jewish	85.6	15,145	84.9	4,251	85.8	10,894
Arab	14.4	2,556	15.1	758	14.2	1,798
	**Mean**	**SD**	**Mean**	**SD**	**Mean**	**SD**
Age	43.4	11.5	40.9	10.4	44.4	11.8
Age at first hospitalization	27.4	9.9	25.7	8.0	28.1	10.5

*COD, co-occurring disorders of chronic psychotic disorder and substance use disorder; non-COD, chronic psychotic disorder (schizophrenia or schizoaffective disorder) without co-occurring substance use disorder*.

Regarding receipt of rehabilitation services, 63% of non-COD patients and 56% of those with COD received rehabilitation services at some point during 2001 and 2016 (*p* < 0.0001). In both groups, an increase over time was observed. Among patients without COD, 35% received rehabilitation services in Period_1_ and this rate increased to 56% in Period_2_, and among patients with COD the rehabilitation service rate increased from 30 to 48% across the two time periods (Table 2).

**Table 2:** Percent of patients who received housing and/or vocational rehabilitation services by period (2001–2009, 2010–2016) and overall, and by COD status.

		**Period**		
**Service**	COD-status^*^	**2001–2009**	**2010–2016**	**2001–2016**
Housing and Vocational	Non-COD	35	56	63
	COD	30	48	56
	Total	31	54	61
Housing only	Non-COD	20	35	41
	COD	18	32	38
	Total	20	34	40
Vocational only	Non-COD	26	44	51
	COD	22	40	46
	Total	25	43	49

*COD, co-occurring disorders of chronic psychotic disorder and substance use disorder; non-COD, chronic psychotic disorder (schizophrenia or schizoaffective disorder) without co-occurring substance use disorder*.

* *p < 0.005 for all COD vs. non-COD comparisons*.

As seen in Table 3, the average duration of housing and vocational rehabilitation services was shorter for COD patients than non-COD patients. It is worth noting that the mean duration of rehabilitation among those who received services for more than 1 year was 2–6 years (792–2223 days), while among those who received services for <1 year the mean duration was 4–6 months (132–191 days). Duration of rehabilitation was inversely associated with percent of hospitalizations with a SUD diagnosis (*p* < 0.001).

**Table 3:** Average duration of rehabilitation (in days) by period (2001–2009, 2010–2016) among recipients of housing and vocational rehabilitation services.

**Service**	**Period**	**Rehabilitation duration (days)**	**non-COD**	**COD**	
			**Mean (SD)**	**Mean (SD)**	***p*-value**
Housing	2001–2009	≥365	2223 (1338)	1754 (1152)	<0.0001
		<365	191 (100)	178 (100)	0.105
		Total	1916 (1432)	1373 (1209)	<0.0001
	2010–2016	≥365	1448 (682)	1305 (659)	<0.0001
		<365	187 (104)	181 (102)	0.28
		Total	1219 (787)	1013 (753)	<0.0001
Vocational	2001–2009	≥365	1104 (643)	916 (532)	<0.0001
		<365	141 (105)	132 (103)	0.06
		Total	704 (687)	459 (523)	<0.0001
	2010–2016	≥365	839 (346)	792 (324)	<0.0001
		<365	146 (106)	132 (100)	<0.0001
		Total	508 (433)	406 (394)	<0.0001

Overall, non-COD patients also received vocational rehabilitation for longer duration than COD-patients. 43% of people without COD who received vocational rehabilitation for more than a year in both periods, compared to 28% of people with COD (Table 3). Also, 18% of non-COD people received vocational rehabilitation for less than a year in both periods, compared to 28% of people with COD.

**Figure 1:**
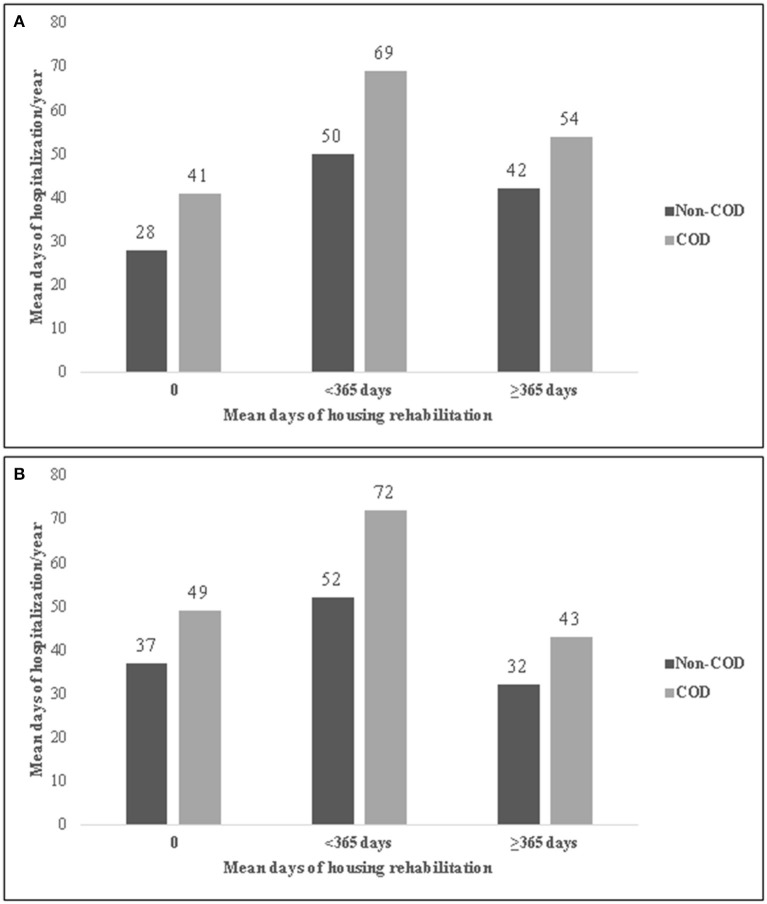
Mean psychiatric hospitalization days per year by duration of housing rehabilitation in Period_1_ (2001–2009) **(A)** and Period_2_ (2010–2016) **(B)**, among hospitalized persons with co-occurring disorders (COD) of chronic psychotic disorder and SUD, Israel, 2010–2016. **(A)** Period_1_. **(B)** Period_2_.

**Figure 2:**
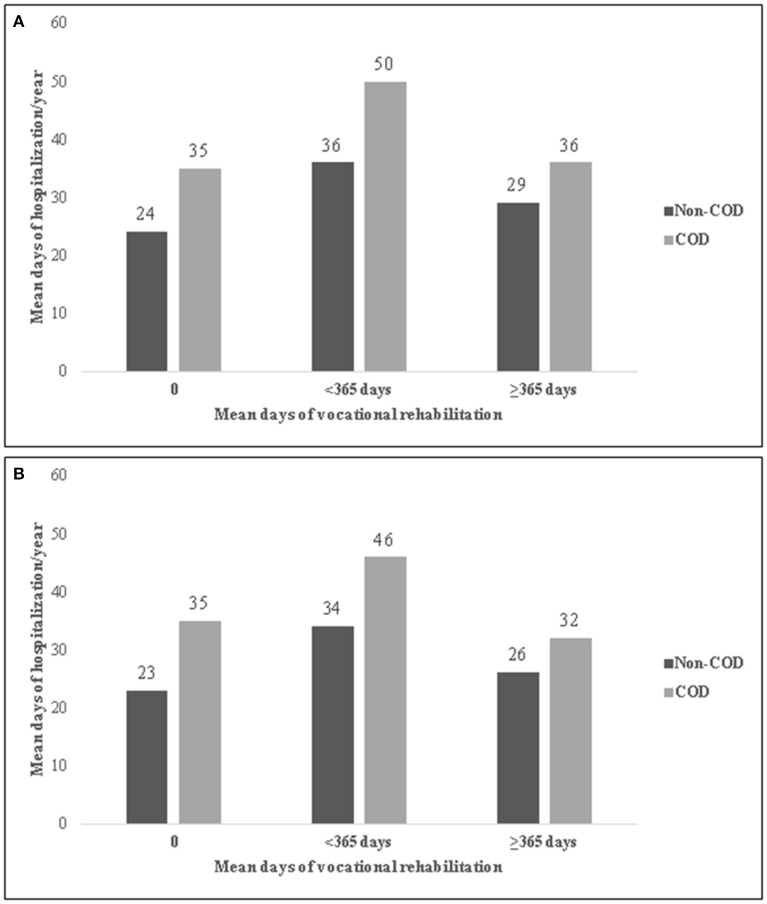
Mean psychiatric hospitalization days per year by duration of vocational rehabilitation in Period_1_ (2001–2009) **(A)** and Period_2_ (2010–2016) **(B)**, among hospitalized persons with co-occurring disorders (COD) of chronic psychotic disorder and SUD, Israel, 2010–2016. **(A)** Period_1_. **(B)** Period_2_.

Among persons who received housing-rehabilitation both during Period_1_ and Period_2_, duration of rehab was inversely associated with annual number of hospitalization days. As seen in Figures 1, 2, the mean number of hospitalization days was highest for those who received housing or vocational rehabilitation services for <1 year. This was true for those with and without COD. In Period_1_ the mean number of hospital days was lowest among those who did not receive any rehabilitation, whereas in Period_2_ those who received more than 1 year of rehab experienced the fewest hospital days per year. COD-status was also significantly associated with hospitalization days—the mean number of hospitalization days/year was consistently higher among those with COD. Comparing Panel A and Panel B, a decrease in hospitalization days from Period_1_ to Period_2_ was noted only among those who received rehabilitation over a year. People who received vocational rehabilitation experienced fewer hospitalization days than people with housing rehabilitation (Figures 1, 2).

**Figure 3:**
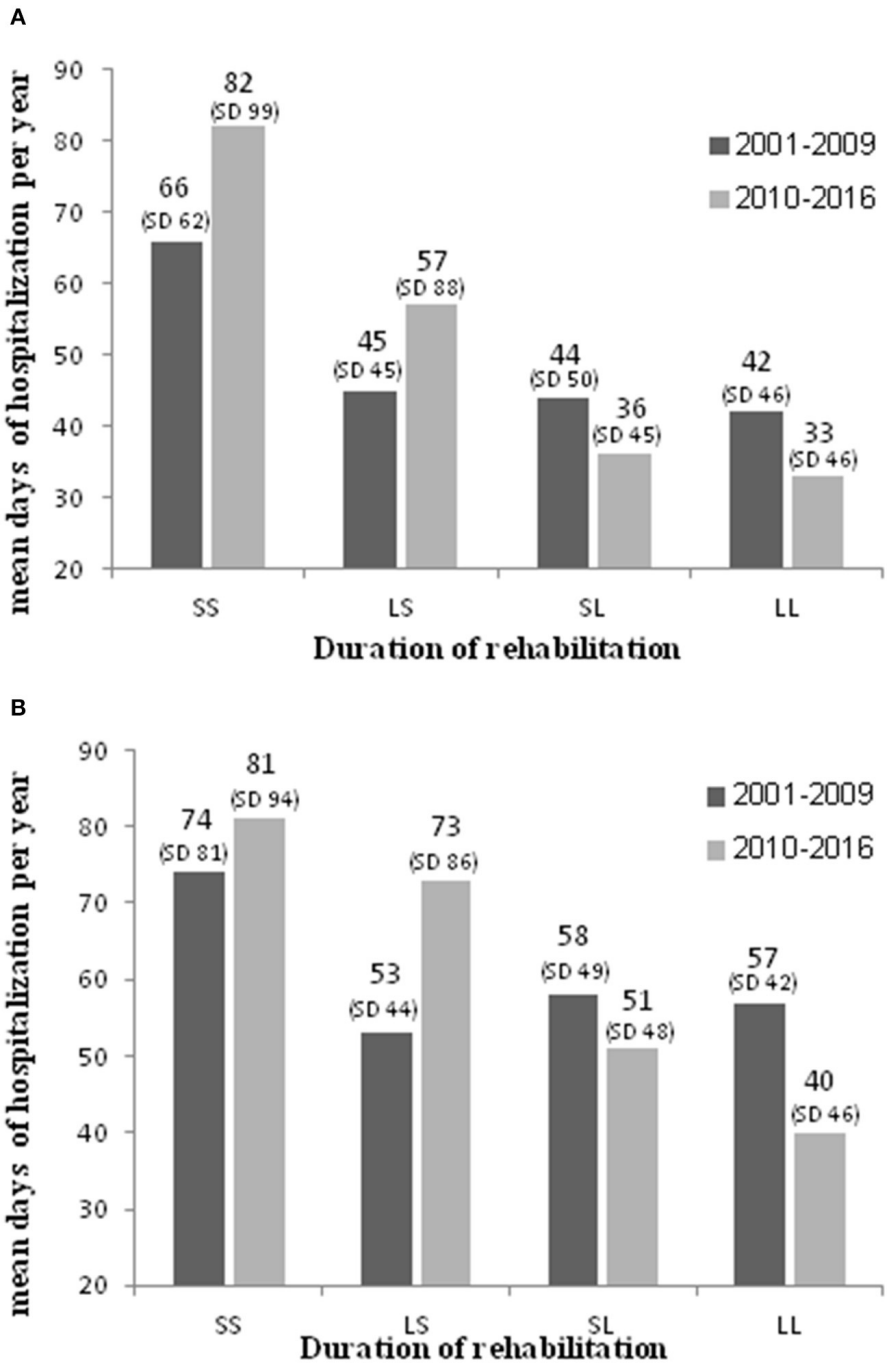
Mean psychiatric hospitalization days per year, by duration of housing rehabilitation in Period_1_ (2001–2009) and Period_2_ (2010–2016), among hospitalized persons with chronic psychotic disorder without co-occurring SUD **(A)** and with SUD **(B)** who received any housing rehabilitation, Israel, 2010–2016. **(A)** Hospitalized persons with chronic psychotic disorder without SUD. **(B)** Hospitalized persons with chronic psychotic disorder with SUD. SS, short rehabilitation in Period_1_ and Period_2_; LS, long rehabilitation in Period_1_ & short in Period_2_; SL, short rehabilitation in Period_1_ & long in Period_2_; LL, long rehabilitation in Period_1_ and Period_2_.

As seen in Figure 3, when we restricted the analysis to those who received any housing rehabilitation in both periods, for both groups, longer rehabilitation (more than 1 year) in both periods was associated with the fewest hospitalization days, and short rehab (<1 year) with the most hospitalization days.

People without COD received housing rehabilitation for longer periods−79% of people without COD who received housing rehabilitation, received rehabilitation for more than a year in both periods, compared to 68% of people with COD. Also, 2% of people without COD received housing rehabilitation for less than a year in both periods, compared to 6% of people with COD (*p* < 0.0001).

**Figure 4:**
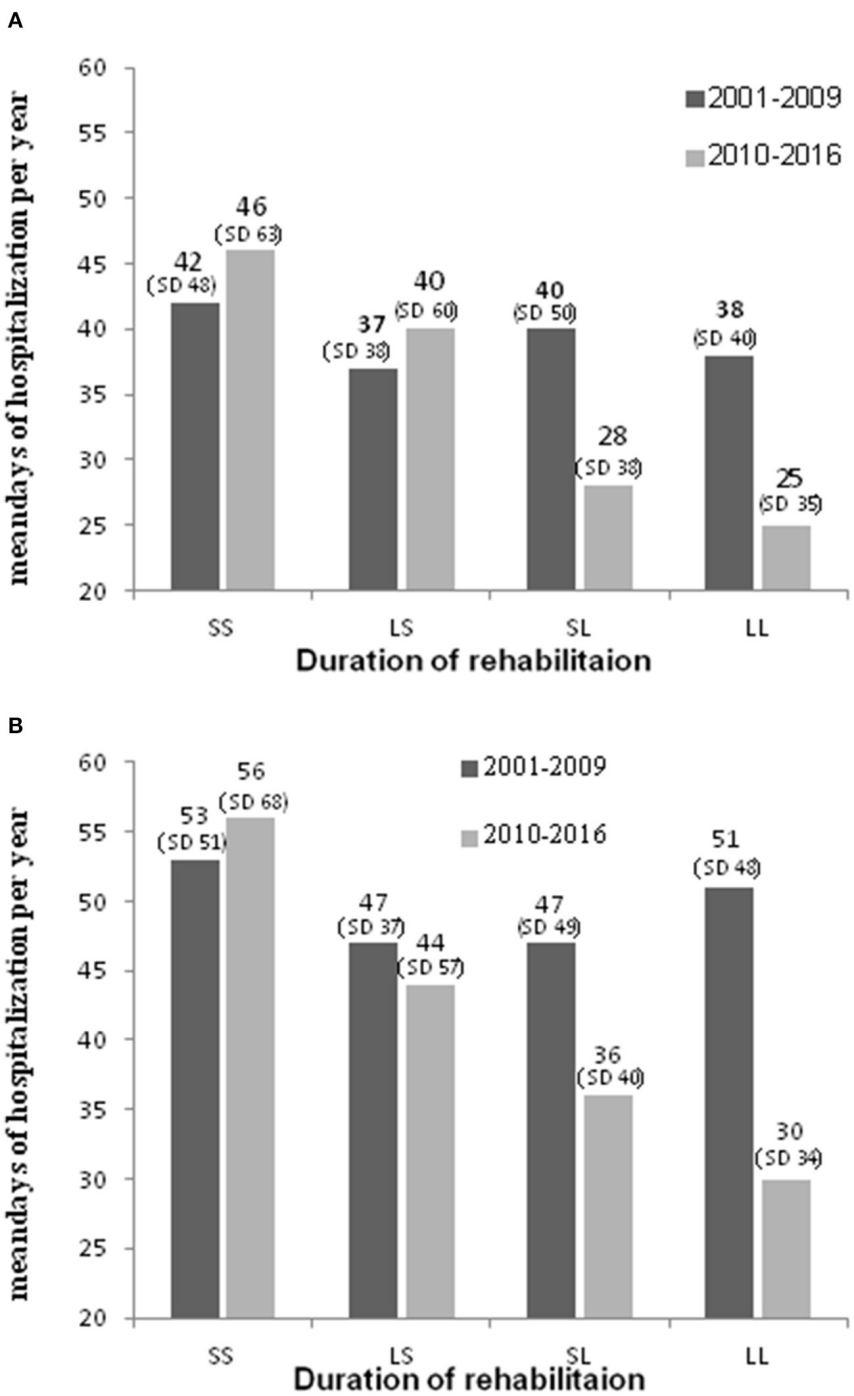
Mean psychiatric hospitalization days per year, by duration of vocational rehabilitation in Period_1_ (2001–2009) and Period_2_ (2010–2016), among hospitalized persons with chronic psychotic disorder without co-occurring SUD **(A)** and with SUD **(B)** who received any vocational rehabilitation, Israel, 2010–2016. **(A)** Hospitalized persons with chronic psychotic disorder without SUD. **(B)** Hospitalized persons with chronic psychotic disorder with SUD. SS, short rehabilitation in Period_1_ and Period_2_; LS, long rehabilitation in Period_1_ & short in Period_2_; SL, short rehabilitation in Period_1_ & long in Period; LL, long rehabilitation in Period_1_ and Period_2_.

As seen with housing rehabilitation, duration of vocational rehabilitation for patients who received any vocational rehabilitation in both periods was also inversely associated with mean days of hospitalization per year (*p* < 0.0001). Having received vocational rehabilitation for <1 year in either Period_1_ or Peroid_2_ was associated with a greater number of hospitalization days per year in 2010–2016 (Figure 4).

People without COD received vocational rehabilitation for longer periods−43% of people without COD who received vocational rehabilitation received rehabilitation for more than a year in both periods, compared to 28% of people with COD. Also, 18% of non-COD people received vocational rehabilitation for less than a year in both periods, compared to 28% of people with COD (*p* < 0.0001).

The association between mean number of hospital days and rehabilitation remained significant upon controlling for age, sex, COD group, percent of hospitalizations with SUD, and age at first hospitalization. Multivariate ANCOVA modeling revealed a significant independent association between mean hospitalization days per year during 2010–2016 and COD-status (*p* < 0.0001). Numer of hospital days was independently associated with percent of hospitalizations with SUD (*p* = 0.025) and with male sex (*p* < 0.0001), and inversely associated with age at first hospitalization (*p* < 0.0001). Age at time of the study was not associated with annual number of hospitalization days.

## Discussion

The results show that from 2001–2009 to 2010–2016 there was an almost 2-fold increase in the proportion of people with chronic psychotic disorder who received rehabilitation. This trend is similar among COD and non-COD patients. In the latter period, two-thirds (63%) of persons without COD and slightly over half (56%) of those with COD received rehabilitation services. The increase in the proportion of people receiving rehabilitation services reflects the development of the rehabilitation system in Israel, which over the years has recognized the need to expand rehabilitative care in the community, thus opening more rehabilitative frameworks. These findings are consistent with previous reports regarding the growth of community rehabilitation services in the country (31, 37).

The percentage of people without COD who qualify for rehabilitation services was significantly greater than those with COD, although the difference is not large. We expected, in light of our clinical experience, a much larger disparity in rehabilitation eligibility in favor of persons without COD. In addition, until recently, and during most years of the current study, there was a declared policy of the Rehabilitation Committees of not providing services to people with active drug use (32). Several explanations for the discrepancy between our hypothesis and the results can be posited. One possible explanation is a cognitive bias which appeared in cases where the Rehabilitation Committee rejected a request for rehabilitation services, due to drug use history. This aroused negative feelings such as disappointment among therapists and patients, and thus such cases might be more recalled than cases where approval was obtained.

Another possibility of the described discrepancy is related to the definition of the COD group in our study (people with at least two hospitalizations, or at least 20% of their hospitalizations with an indication of SUD). We found that people from the COD group who received rehabilitation services, had SUD recorded in less than half of their hospitalizations. Approval for rehabilitation services might have been granted during hospitalizations without active SUD, whereas in other hospitalizations with active drug use, rehabilitation services were refused.

In addition, hospital therapists who submit the patient to the Rehabilitation Committee may not always fully disclose drug use information when the use is not very intense (even in the presence of SUD diagnosis), out of a concern that the information would cause the Committee to reject the request for rehabilitation services.

Our results indicate that persons who received rehabilitation for a longer duration (a year or more) in Period_1_ and/or Period_2_, had on average fewer hospitalization days annually in Period_2_ than those who received rehabilitation for <1 year. This finding corresponds with worldwide studies, including in Israel, which show that community rehabilitation services are associated with fewer hospitalization days and less frequent hospitalizations among people with schizophrenia (20–23).

The finding that people who did not receive rehabilitation services had fewer days of hospitalization than people who received rehabilitation is surprising at first glance. A possible explanation for this could be better underlying clinical condition or stronger support from family or other sources, which enable these persons to manage in the community without rehabilitation services. Indeed, the group who did not receive services includes a small proportion (<10%) whose application for rehabilitation services was refused.

The finding that those who received rehabilitation services for a short duration had, on average, more hospitalization days compared to people with longer rehabilitation, can be easily understood. People who dropped out from the rehabilitation setting may have been discharged from the hospital before a sufficient improvement was achieved and thus were not yet able to adapt to a rehabilitation community setting. It is also possible that joining a new therapeutic framework in the community posed a stressor they could not easily overcome, thus leading to a clinical exacerbation and re-hospitalization. Another explanation could be that some clinical or personality characteristic made it difficult for them to cope with the rehabilitation requirements and to benefit from the rehab services, and these same characteristics had an impact on the higher frequency of hospitalizations and greater number of hospitalization days per year.

The relatively low number of hospitalization days among people receiving longer-duration rehabilitation reinforces the assumption that prolonged rehabilitation is beneficial to patients' mental health and helps prevent re-hospitalization. This assumption is further supported by the finding that persons who received rehabilitation for a short time in Period_1_ and then received longer rehabilitation in Period_2_, experienced fewer days of hospitalization. At the same time, the possibility of an underlying confounding clinical or personality characteristic, as mentioned, or higher levels of intrinsic motivation and better family relationship (38) cannot be ruled out.

Duration of vocational rehabilitation, as well as housing rehabilitation, was found to be inversely associated with number of hospitalization days. Although we cannot assume independence between housing rehabilitation and vocational rehabilitation, as some may receive both, those who received vocational rehabilitation experienced fewer hospitalization days per year than those who received housing rehabilitation. Vocational rehabilitation, therefore, may have an even greater effect than housing rehabilitation on the patient's mental condition and on hospitalizations. Employment is especially important for people with COD, because it reduces a sense of emptiness by filling their time with productive activity, improves their quality of life and provides a sense of meaning and self-esteem. This may in turn lessen the emotional need to return to substance abuse and reduce the risk of relapse and re-hospitalizations (39, 40).

We previously reported that from 1991–2016 there was no improvement in the average number of days of hospitalization per year for people with COD, while for those without COD the number of hospitalization days was reduced by half (20). Nonetheless, the most striking results of the present study are that rehabilitation is at least as beneficial for people with COD as for those without COD, and this is also reflected in a decrease in hospitalization days for both groups. This, despite the fact that at the time of the study there were very few rehabilitation frameworks designed for COD patients, and therefore most chronic psychotic people with SUD underwent rehabilitation in settings that did not have COD specialty. However, this interpretation is posited with caution, since it is likely that non-specialized rehabilitation settings are most suitable for persons with lower severity of SUD, such as occasional drug abuse, while those with severe addiction are less likely to manage in a non-specialized setting.

The significantly higher annual number of hospital days, on average, among persons with co-occurring chronic psychotic disorders and SUD, even after controlling for age, sex and percent of hospitalizations with SUD and age at time of study, and the higher frequency of hospitalizations (20), may be a result of more frequent substance-related exacerbations of the clinical condition (8, 41), greater difficulty in cooperating with treatment over time, and longer periods of non-adherence to treatment, as compared with non-COD patients (11, 41–43).

It is also important to note that compared with those without COD, the COD group who received rehabilitation in 2001–2009 and 2010–2016, had a 2–3-fold higher proportion of people who received rehabilitation for less than a year. This might be attributed to personal characteristics of some persons with COD, such as impulsivity (44–46), or directly to drug use that led to the cessation of rehabilitation services, and/or to a shortage of rehabilitation frameworks specifically designed for COD patients.

## Strengths and Limitations

This study utilized data from the Israel Mental Rehabilitation Register and the National Psychiatric Case Register that captures virtually all psychiatric hospitalizations. Complete hospitalization histories were obtained for all in-patients diagnosed with schizophrenia or schizoaffective disorder in Israel in the period 2010–2015. The retrolective design of the study precluded the analysis of some important demographic and clinical variables, such as severity of the psychotic disorder and SUD, level of functioning, degree of motivation for rehabilitation and level of family support. These factors affect the ability to sustain and benefit from rehabilitation services and on the likelihood of re-hospitalization. Because the severity of patients' SUD was unknown, it was not possible to ascertain whether SUD severity was related to being accepted into and staying in a rehabilitation framework. The relationship between the severity of SUD and the number of hospitalization days is also unknown. It is also possible that for some COD patients, SUD developed subsequent to their first hospitalization, or that SUD was less documented in earlier years. However, we found that for the majority of COD patients, SUD was already documented early in their hospitalization history. Specifically, 85% of patients hospitalized in both periods (2001–2009, 2010–2016) were already diagnosed with SUD in Period_1_. We believe therefore, that the cross-period comparisons are trustworthy, albeit perhaps with some margin of bias.

In addition, in our study, COD is defined as a SUD diagnosis present in at least two hospitalizations or in at least 20% of each patient's hospitalizations rather than the more commonly used “lifetime” or first-hospitalization SUD definition. We believe this enhances the COD diagnostic validity (specificity) as it describes a chronic comorbid drug use pattern. Indeed, on average, a SUD diagnosis was documented in more than half (54%) of all hospital admissions amongst those classified as COD. We did not adopt stricter COD criteria (i.e., SUD diagnosis appears on a greater proportion of admissions) out of a concern that the secondary SUD diagnosis may be under-recorded, as has been noted in other countries (47). Furthermore, since the standard urine drug tests used in Israel do not detect commonly-used novel psychoactive substances (NPS), suspected drug use cannot always be confirmed in the event that the patient denies use.

Twenty-nine individuals with more than 80 hospitalizations were not included in the analyses. The decision to exclude them stemmed from an impression that the excessive numbers of hospitalizations might have been due to double reporting. Regrettably, the data for these individuals is unavailable and we are unable to rerun the analyses with them included to assess the impact of the exclusion. We believe the impact is negligible given the small number excluded.

Lastly, the unequal duration of Period_1_ (2001–2009) and Period_2_ (2010–2016) may introduce some measure of confounding by factors differentially distributed across the two time periods. The periods were defined in accordance with the study objective to assess the government's decision to allocate additional resources toward the treatment and rehabilitation of persons with COD. We are unaware of any substantial temporal changes in treatment or quality of services, beyond the expansion of rehabilitation services. Defining the unit of analysis for number of hospital days as “per year” rather than per period, will have minimized the effect of any such confounding.

## Conclusions

In summary, the results of our study show that people with COD appear to have the potential for significant rehabilitation when given the opportunity. Also, it seems that at least some people with COD can go through a prolonged rehabilitation process in the community even in settings that are not specifically designed for people with SUD. However, persons with COD are less likely to remain in rehabilitation for prolonged periods. Findings of this study show that when rehabilitation is prolonged and lasts for at least a year, there is a significant reduction in hospitalization days. These findings reinforce the clinical importance of sustained rehabilitation for people with COD, for whom longer rehabilitation may be required to commit to rehabilitation and to attain clinical stabilization than for persons without COD. It is likely that if rehabilitation frameworks are dedicated for people with COD, those with even more severe SUD may benefit from rehab and will be less likely to drop out early. The process of expanding the rehabilitation services designed for COD patients should be accelerated and more healthcare providers should be trained in evidence-based practices for COD. As this process continues to evolve, we will likely see a significant improvement in the clinical condition of these patients over time and in their ability to manage and re-integrate in the community, and as a result their need for hospitalizations is expected to decline.

## Data Availability Statement

The datasets analyzed in this study were obtained from the Ministry of Health of Israel. Access to the data is highly restricted due to the sensitive nature of pyschiatric patient data. Requests to access these datasets should be directed to Mr. Reuven Eliyahu (reuven.eliahu@moh.gov.il) Data Security Office, Ministry of Health.

## Author Contributions

All the authors designed the study. SF wrote the first draft of the manuscript. TB-A designed the methods for data analysis. YN and PR proofed and revised the manuscript. All authors contributed to the article and approved the submitted version.

## Conflict of Interest

The authors declare that the research was conducted in the absence of any commercial or financial relationships that could be construed as a potential conflict of interest.
